# Comparison of the ArtekMed mixed reality teleconsultation system with a standard video call system in critical care: user acceptance and feasibility analysis

**DOI:** 10.1186/s40635-025-00758-4

**Published:** 2025-05-08

**Authors:** Nadine Liebchen, Julia Schrader-Reichling, Frieder Pankratz, Marc Lazarovici, Selina Kim, Jennifer Tempfli, Ulrich Eck, Stephan Prückner

**Affiliations:** 1 Institut Für Notfallmedizin und Medizinmanagement (INM), LMU Klinikum, LMU München, Munich, Germany; 2 Department of Anaesthesiology, LMU University Hospital, LMU Munich, Munich, Germany; 3 Chair for Computer Aided Medical Procedures and Augmented Reality, Faculty of Informatics, Technical University of Munich, Munich, Germany

**Keywords:** Mixed reality, Augmented reality, Virtual reality, Teleconsultation, Telementoring, Intensive care, ArtekMed, Future of intensive care

## Abstract

**Background:**

Telementoring and teleconsultation are increasingly employed for collaboration within the healthcare system. The ArtekMed alliance project has developed a mixed reality (MR) teleconsultation system for intensive care units (ICU) using virtual reality (VR) and augmented reality (AR), facilitating real-time interaction between the real world and its reconstructed virtual model, shared by two or more coworkers.

**Objective:**

We aimed to explore the feasibility and user acceptance of the ArtekMed MR teleconsultation system in a critical care setting and compare it to a standard teleconsultation system using a simulated video call.

**Method:**

A randomized cross-over study was conducted in a local simulation center: A remote expert (VR user) solved four clinical scenarios, each involving the treatment of an ICU patient with respiratory failure in collaboration with a local practitioner as facilitator (AR user). They used either the MR system (intervention) or a simulated video call (control). A mixed-methods approach was followed to explore structured pre- and post-trial interviews with qualitative and quantitative analyses including standardized usability scores (NASA Task Load Index, System Usability Scale SUS).

**Results:**

Twenty-five professionals with intensive care experience completed 100 simulated scenarios. The ArtekMed system achieved an average SUS score of 66, while the simulated video call system was rated almost excellent (SUS score: 84). In three out of four scenarios, the perceived workload using the MR teleconsultation system did not significantly differ from the workload using the standard video call. Most users rated working with both teleconsultation systems positively and anticipated increased efficiency and feasibility with greater familiarity with the MR system. Common issues included visual impairment due to insufficient graphical resolution and unfamiliarity with handling the equipment. 80% of the participants expressed willingness to incorporate the system into their ICU work.

**Conclusion:**

Collaboration in the ICU using a real-time MR teleconsultation system was rated as a promising technology by the majority of the participants for future use. Technical imperfections seem to prevent further implementation at this stage. Thus, the MR reconstruction needs improvement before clinical implementation.

**Graphical abstract:**

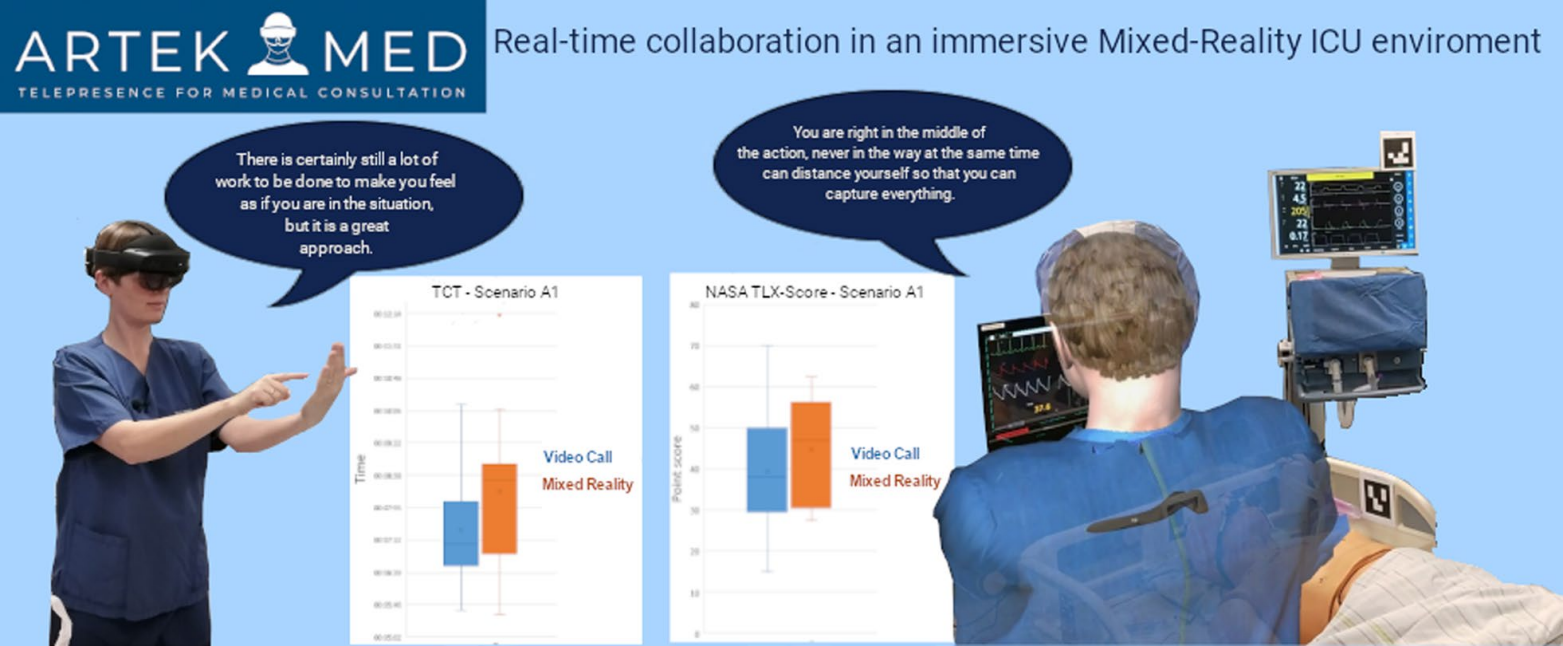

**Supplementary Information:**

The online version contains supplementary material available at 10.1186/s40635-025-00758-4.

## Introduction

Numerous studies have demonstrated the positive effects of using telemedicine in intensive care units: reductions in mortality, shorter ICU stays, fewer patient transfer transports, fewer invasive ventilations, and improved adherence to intensive care quality goals [1] have been reported in large multicenter studies [2–5]. Using existing teleconsultation systems, the interaction between coworkers is currently limited to speech and sometimes supported by a video signal [6]. One way to tackle these limitations of telephone or video signal-supported consultation, such as two-dimensionality, lack of interaction possibilities, and restricted field of view, is by utilizing virtual reality (VR) and augmented reality (AR) technology.

In AR, the user usually sees and interacts with the real world, while perceiving additional, mostly visual but also possibly auditory or olfactory, virtual cues in the environment. Virtual reality provides a completely artificial surrounding with different degrees of immersion for the user. The combination of real and virtual elements is called mixed reality (MR). Extended reality (XR) is used to describe technologies ranging from AR to VR. In this paper, we use the terms XR and MR interchangeably.

Although XR applications are increasingly used in the healthcare system [7–9], Bruno et al. stated that there is a need for further studies exploring the applicability in patients’ treatment.

XR solutions can support physicians performing complex procedures in critically ill patients: AR provides additional images to improve needle tracking while placing a central line [7, 10] or to stabilize the physician’s view on the surgical field while performing a percutaneous dilatational tracheostomy [11].

These applications provide additional information to the physician but do not offer the possibility to share it with coworkers or interact with them in a virtual or augmented environment.

Dinh et al. collected common AR features of MR solutions that allow interaction between AR and VR users: e.g. virtual annotations of the VR user visible in the real surroundings or digital overlays of 2D/3D images, for instance, the remote VR user’s hands or tools, in the AR user’s view [12]. Immersive VR technique was, for example, successfully tested for the treatment of auditory verbal hallucinations of schizophrenia patients via live interaction with an avatar—a virtual personification of the hallucinations’ origin, designed after the patient’s imagination [13].

To respond to the specified needs, MR teleconsultation or telementoring systems for the medical sector, using full immersive MR extending the real-time interaction possibilities between the remote and the local communicator, are currently being developed [14–18]. Gasques et al. developed ARTEMIS, a real-time MR collaborative system, for telementoring surgical procedures including the virtual reconstruction of the surgical field. The STAR platform developed by Rojas-Muñoz et al. is a surgical telementoring platform using AR on the mentee’s side and providing a top-down camera perspective of the surgical field for the remote expert. Both systems allow the remote expert to interact via digital annotations with the AR user.

To increase the level of immersion of the remote expert, the ArtekMed alliance project [19] began developing a 3D telepresence system applicable to medical emergencies [16] where the virtual reconstruction is not limited to the medical area of interest. Further development led to a system for MR teleconsultation to support wards at an intensive care unit [15]. Through ArtekMed, remote experts can join a local 3D reconstructed ICU in VR and support local coworkers equipped with an AR system in real-time.

Following a pilot study and two clinical trials with a small number of cases [16, 20], this paper describes the comparison of the ArtekMed MR teleconsultation system to a simulated video call teleconsultation system used in a critical care setting. Our objectives were to identify possible differences between these systems regarding user acceptance, feasibility, and time needed for medical interventions.

## Methodology

This study was approved by the ethics committee of the Ludwig-Maximilians-University Munich (LMU) (project number 22-0882).

### Study design

A simulation-based, mixed-methods randomized cross-over trial was conducted in the Human Simulation Center (HSC) of the Institut für Notfallmedizin und Medizinmanagement (INM), LMU University Hospital. The study protocol can be seen in Table 1. Four medical scenarios that are likely to happen during the daily work routine of healthcare professionals at an ICU were developed to evaluate the interaction between an inexperienced nurse (facilitator) at an ICU, contacting a medical specialist not at the site (remote expert, test subject) for help. The simulated medical scenarios were complex (see Additional File A), which created the necessity for a skilled moderator as a facilitator, following a structured script (see Additional File B) to moderate the scenarios. All participants used the test system (MR) and the control system (simulated video call via intercom system) twice, completing all four simulated scenarios. Based on the experience from our previous study [20], four different medical scenarios were used instead of two identical scenarios to be completed twice, to avoid the effects of habituation.

**Table 1:** Study protocol

Pre-session		
Questionnaire	Anonymization of participants, age, gender	
Structured interview	Profession, current job setting, general usage of telecommunication systems, acceptance of technical innovations [21], previous experience with VR/AR telecommunication systems, expectations concerning the usability of VR/AR consultation in healthcare practice	
Controlled experiment		
Scenario	Teleconsultation method	Standardized questionnaire
A1 “Atelectasis “	MR/video call	NASA-TLX or NASA-TLX and System Usability Score (SUS)
A2 “Dislocation of endotracheal tube”		
B1 “Accidental extubation “		
B2 “Tension pneumothorax “		
Post-session		
Structured interview	General user satisfaction, specific operational aspects of the system, (dis)advantages, possible future applicability	
Target population	Healthcare professionals	
Inclusion criteria	Working experience in an ICU; binocular vision	
Recruitment	Addressing ICU workers of local and regional hospitals via social media, handouts and email	

A cross-over design was implemented to consider the possible impact of the scenario order on the outcomes. The order of the scenarios and the telecommunication system were randomized using computerized random numbers.

Taking into account the exploratory nature of the study, we aimed to recruit at least 16 participants (4 scenarios × 2 telecommunication systems x at least 2 participants for each kind of sequence).

### Qualitative and quantitative methods

#### Outcome

The primary outcome was user acceptance, measured via the NASA-TLX [22], the SUS [23], and the post-session interview (Additional Files C–E). Secondary outcomes were efficiency, defined as the time needed to complete a scenario, and feasibility, derived from the post-session interview.

#### Qualitative methods

For the qualitative analysis of the data, an exploratory approach was taken, based on Mayring’s qualitative content analysis method [24] and components of the grounded theory [25]. Two investigators used an inductive coding technique to analyze the structured interviews. A specific focus was placed on the within-subject comparison. The software used to analyze the data was MAXQDA 22 (VERBI Software GmbH).

#### Quantitative methods and statistical analysis

The task completion time (TCT) was measured by coding the beginning and end of each scenario. TCT was treated as continuous data (hh:mm:ss). Periods of solving technical issues related to the telesupport system were subtracted from the TCT. The NASA-TLX score and the SUS are standardized questionnaires providing categorical data. Possible differences regarding perceived workload, usability score, and TCT between the teleconsultation methods were explored using two-sample t tests for each scenario. For all statistical tests, a *p* value of 0.05 was considered statistically significant. Software used to analyze the data were Excel 2016 (Microsoft Corporation), MAXQDA 22 (VERBI Software GmbH), and SPSS Statistics Version 29.0.0.0 (IBM Germany GmbH).

### Technical setup

#### Real-time mixed reality teleconsultation system

**Fig. 1:**
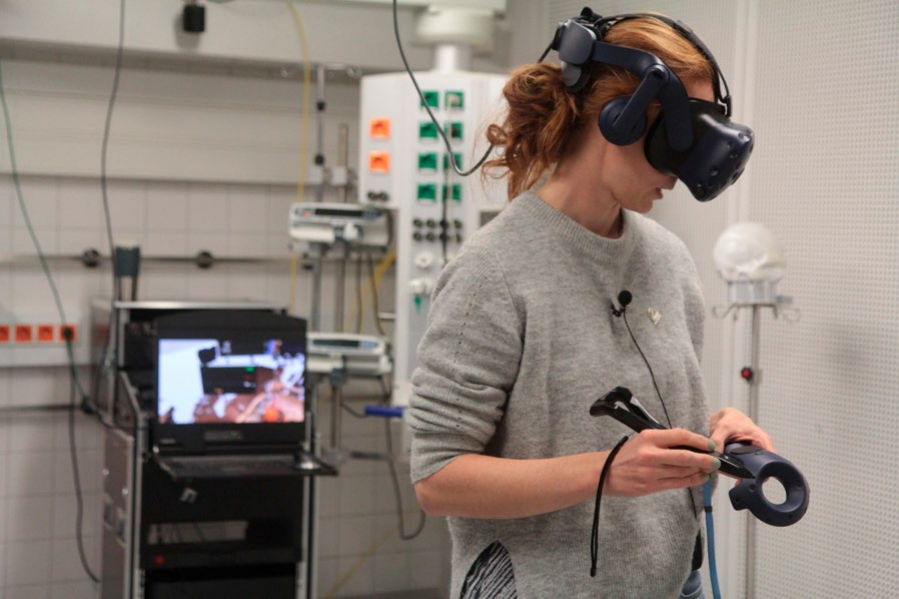
Remote expert with VR HMD, controller, and pencil

The development of the employed telecommunication system is described in detail by Roth et al. [15]. In brief, an immersive MR environment was created, enabling the remote expert to participate in the simulated treatment of the ICU patient (see Additional File F). The scene was virtually reconstructed using the view of five RGB-D cameras (Microsoft Azure Kinect) mounted on the ceiling [26]. To realize the visualization of the scene, Unity3D, a well-known game engine (Unity Technologies, San Francisco) was used. The local staff was wearing Microsoft Hololens 2 AR HMD and the remote expert was equipped with an HTC Vive VR HMD, a standard-issue Vive controller, and a Logitech Ink Pen (Fig. 1).

**Fig. 2:**
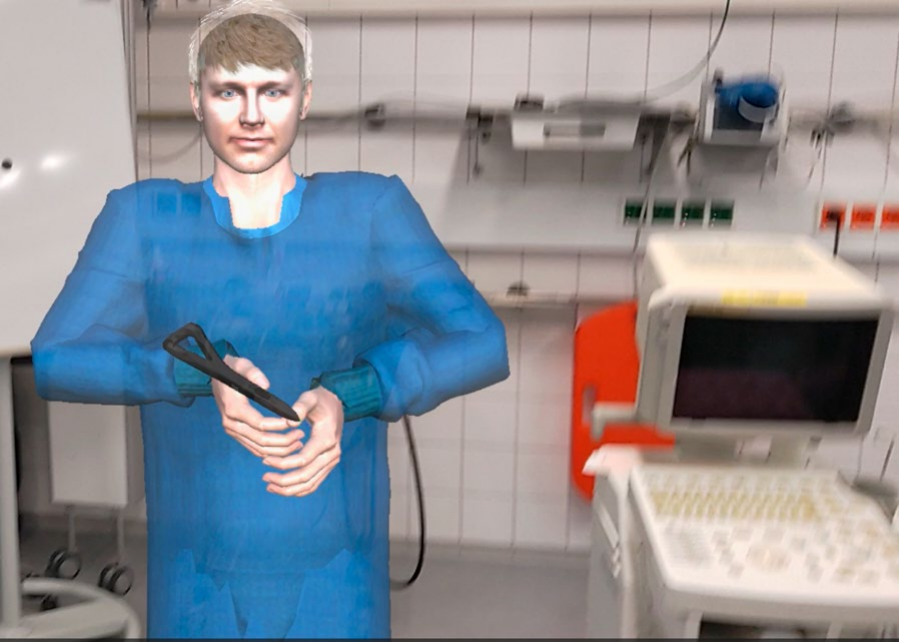
HoloLens 2 AR HMD view: the avatar representing the remote expert

The verbal communication during the intervention was realized using a Skype call. The remote expert joined the AR user incorporated as an avatar [27] (Fig. 2). The screens of the patient monitor and the ventilator monitor were integrated into the VR environment as virtual monitors that could be placed freely by the remote expert. The screens were streamed to the VR user using screen grabbers.

**Fig. 3:**
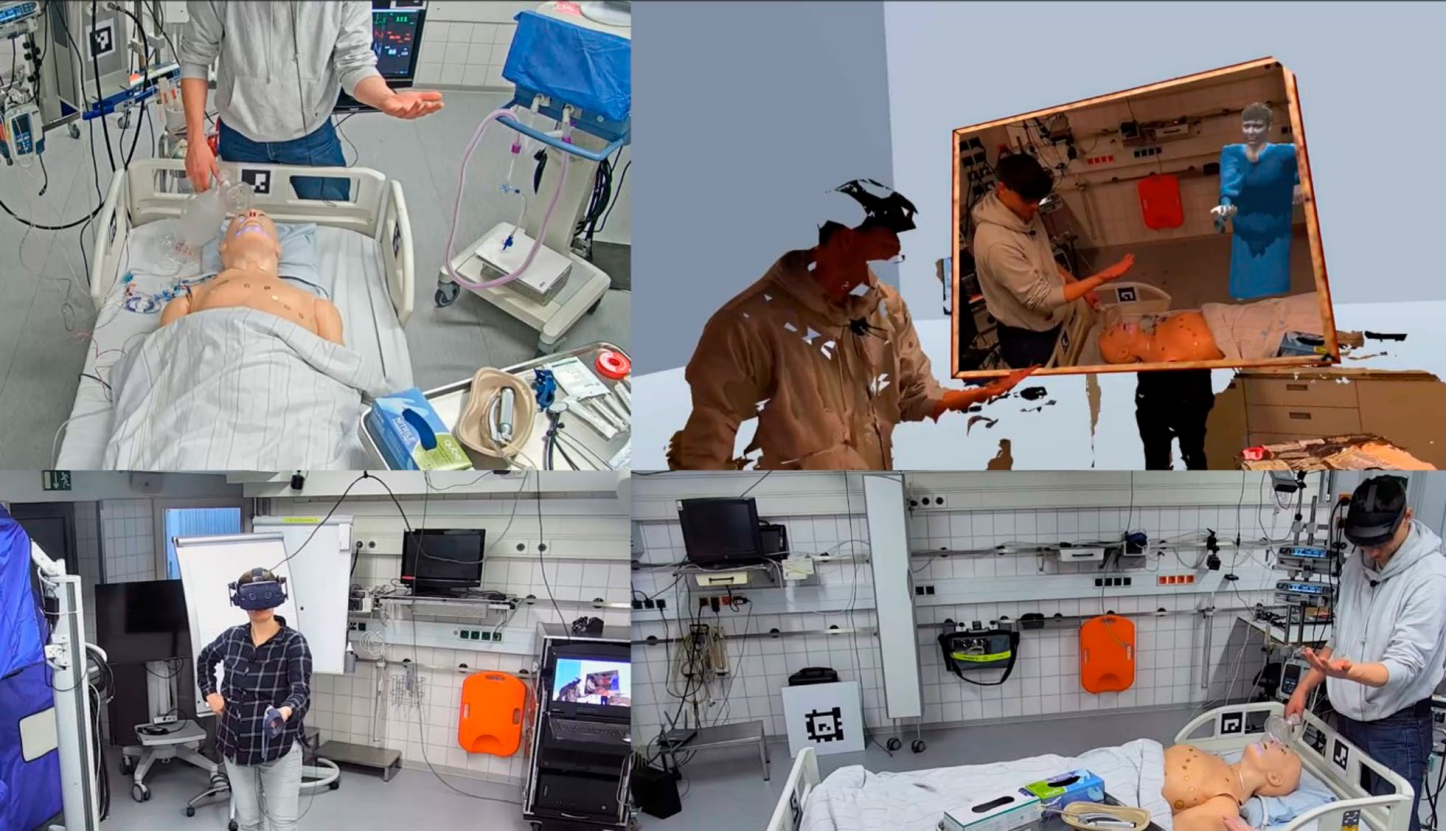
Four perspectives showing the interaction possibilities. The remote expert (bottom left) and the avatar (top right) demonstrate a gesture, which is copied by the AR HMD user

AR and VR users could interact by talking to each other, highlighting and marking respective features in the room or on the patient with the pen or the pointer, and sharing information on virtual screens (patient monitor, ventilator monitor). The Logitech Ink Pen could be utilized both as a pointer and as a pen for making annotations. Using the controller, the VR user was able to access digital patient information and place it freely within the virtual space. To overcome quality limitations in the 3D reconstruction, a digital, projective bisector mirror was developed to ensure better graphical resolution in partial image sections. Activated via the controller, the live image from a single Kinect camera was placed on a plane in the field of view of the camera in such a way that the image appears to be a mirror floating in space [28] (Fig. 3).

#### Simulated video call teleconsultation system

**Fig. 4:**
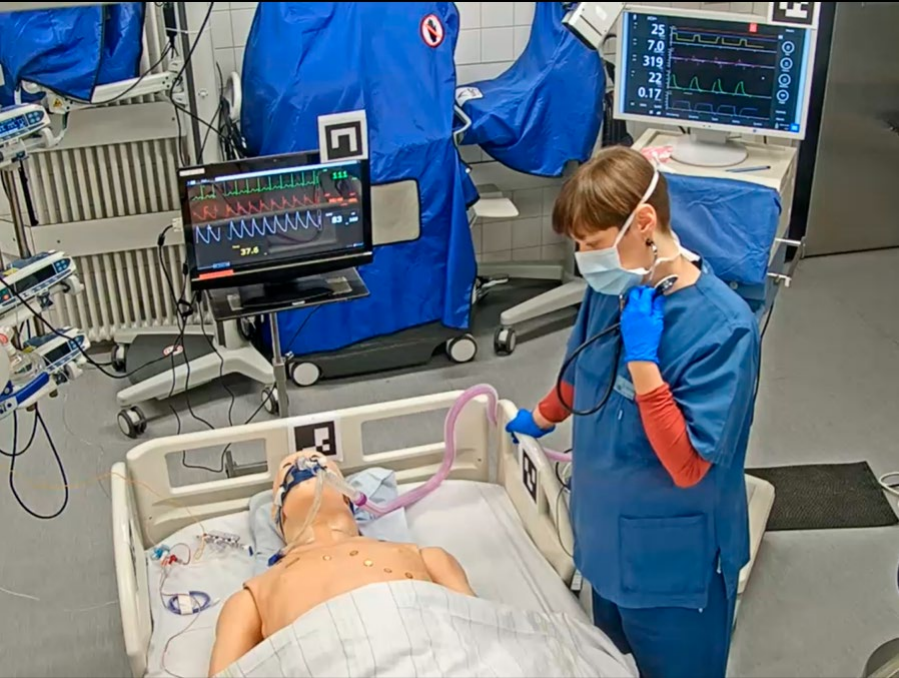
Camera perspective simulated video call

During the simulated video call sessions, the remote expert sat in front of a conventional 2D monitor with 1080p resolution, showing the video stream from a single camera mounted on the ceiling of the simulation room (Fig. 4). The picture was static, without a zoom or remote control function. The audio feed was bidirectional using the HSC’s intercom system.

## Results

### Participants’ characteristics

All participants met the inclusion criteria. In total, 25 healthcare professionals (13 nurses and 12 physicians) took part in the study. All test subjects completed four scenarios, all questionnaires and interviews. There was no missing data. Participants were 32.2 years old on average (SD 7.1 years), ranging from 20 to 47 years, and 15 (60%) were male. The average working experience in an ICU was 6.9 years (SD 6.0 years), ranging from 9 months to 20 years.

Most subjects regularly worked at the patients’ bedside, performing numerous rounds, consultations, or therapies per workday. Fourteen (56%) participants stated they were never or rarely asked for advice without being on the scene, and eight (32%) were regularly or often contacted by coworkers for consultation. Organizational issues, questions concerning the treatment, and case discussions were the most frequent reasons to initiate a consultation. Means of communication frequently used were telephone and smartphone messenger, rarely email or app services.

Twenty-two (88%) participants had heard of “VR or AR technology” before and eleven (44%) participants had already used the technology, but only two of them used frequently. Responding to four questions that gauged their acceptance of technical innovations, the subjects’ mean acceptance was 16.4 out of 20 (SD 3.1).

### Comparison of the teleconsultation systems

During our study, 25 subjects conducted 100 clinical scenarios, of which half were using the MR teleconsultation system. All scenarios were completed, with a focus on the implementation of communication and interaction between the remote expert and the standardized caregiver at the bedside rather than the correct medical treatment of the patient. During the 50 MR scenarios, there were 7 interruptions due to technical problems. Twice, a restart of the Unity software was necessary, and on two occasions, the setup was incomplete requiring a restart of the HoloLens HMD once and activation of the audio communication channel once. In addition, two user errors were resolved through re-instruction. One technical issue was not related to the MR system, but to incorrect settings in the ventilator simulation (Additional File I). These errors led only to minor disruptions of the process but did not cause termination of the scenario.

**Fig. 5:**
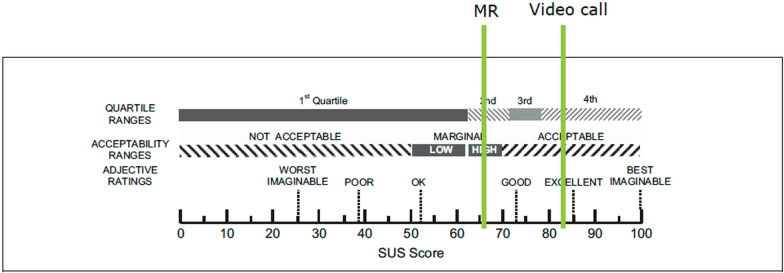
Visualization of SUS results: according to literature reviews, the average SUS score is 68 and the top 10% ratings are represented by a score above 80.3 [29, 30]. Note that although the scale stretches from 0 to 100, it does not represent percentages.

During the post-session interviews, participants generally expressed satisfaction with both teleconsultation systems. Many described the systems as easy to use and stated that the scenarios felt “realistic”, allowing them to act “intuitively”. Some participants mentioned that the MR system initially felt “unfamiliar” or “irritating”, leading them to indicate a preference for the video call system (Table 2). These user comments are supported by the results of the SUS. The participants rated the usability of the video call as “acceptable” (84), above average and the MR teleconsultation system as “high marginal” (66), which is the average on the acceptability range of the SUS (Fig. 5).

**Table 2:** User comments

User comments on feeling of presence and workload using the MR system		
“MR has the advantage of having a consolidated amount of information, and with some routine, one would have an advantage over the video call. The downside to MR compared to video call is that it increases stress levels or cognitive load—possibly due to a lack of familiarity, but it simply requires more training.”		
About the MR system: “You are right in the middle of the action, never in the way and at the same time can distance yourself so that you can capture everything.”		“There is certainly still a lot of work to be done to make you feel as if you are in the situation, but it's a great approach.”
User comments on familiarization time and ease of use		
“It was difficult to remember which buttons to press for what, but that would all come with practice and routine.”	“That’s achievable for anyone.”	“Once you've done it a few times, it works well; very intuitive—and that's even though I have no prior experience with PlayStations or other controllers.”
User comments on the image quality and the digital mirror		
“With the mirror, one could see everything more clearly, but it was still too imprecise. In one scenario, the mirror did not function properly (it was always under the bed). During the thoracic drainage, I could not discern anything on the table; therefore, I had to use the mirror. It helped look at the other side of the bed while inserting the needle into the thorax, but it was still too far away.”		
“The mirror was good because the image was so clear.”	“[I] consistently used the mirror as it provided better image quality.”	"Initially cool, confusing, the popping up was disruptive, not realistic. Better image quality is crucial for usage.“

The users’ subjective TCT did not differ between the two systems: nine (36%) participants estimated that they were faster when using the MR system, eight (32%) favored the simulated video call and eight (32%) were indecisive.

**Fig. 6:**
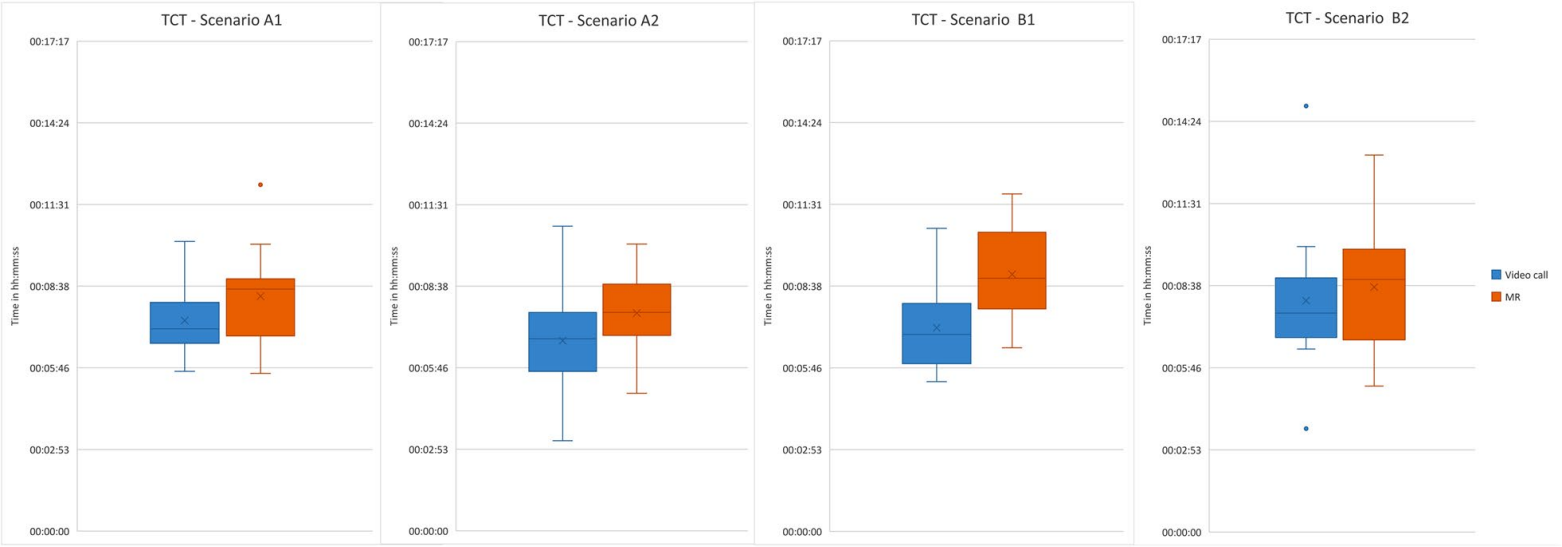
Boxplots of TCT for all scenarios

Accordingly, the quantitative analysis does not indicate a significant difference regarding TCT between the teleconsultation systems, except for scenario B1 (mean MR 09:03 min, mean video call 07:11 min), [*t* (23) = −3.0; *p* = 0.007] (Fig. 6, detailed numbers in Additional File G).

**Fig. 7:**
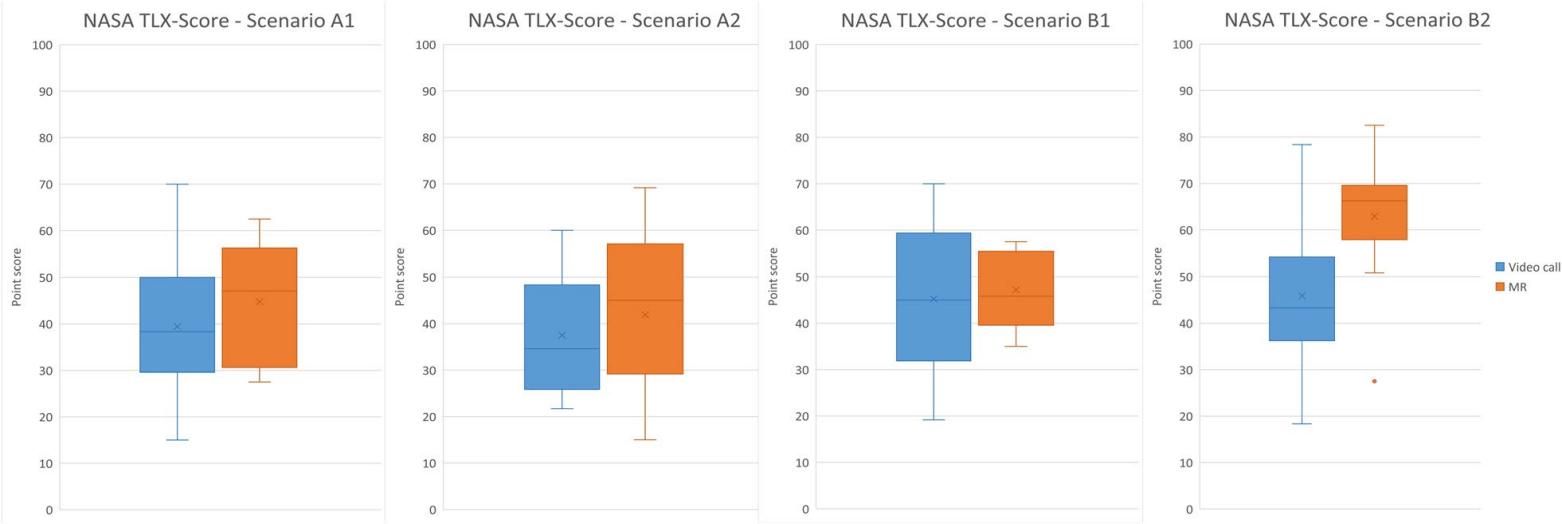
Boxplots of NASA-TLX scores for all scenarios

The NASA-TXL (*N* = 100) does not indicate a significant difference regarding workload between the teleconsultation systems, except for scenario B2 (mean MR = 62.9, mean video call = 45.9), [*t* (23) = −2.9; *p* = 0.008] (Fig. 7, detailed numbers in Additional File H).

During the post-session interviews, users frequently mentioned that the MR system offered independence and commended the additional interaction and communication options. Several participants described the entire setting as realistic and immersive (Table 2). On the other hand, four users (16%) observed that the MR systems would not offer an advantage over the video call if patient information and monitor data were fully accessible in a digital format.

Users reported varying feelings of security and comfort with each system. During the MR sessions, a few mentioned being overwhelmed by a sense of insecurity, and 11 (44%) participants stated that they felt safer with the simulated video call. In contrast, eight (32%) described the MR system to be more secure or equally safe, and six (24%) were indecisive. Several participants noted that the video call conveyed a sense of safety, as it was familiar and required no adaptation period. In addition, nine (36%) users estimated that this would also be the case for MR applications with multiple uses.

During the pre-session interview, 18 (72%) agreed that VR and AR systems would help to improve medical treatments, 7 (28%) were unsure and none of them negated. When asked again after the completion of the study, 21 (84%) were convinced (Additional File J).

Twenty (80%) participants were interested in incorporating the system into their daily work routines, while five (20%) declined.

### Usability of MR teleconsultation system

When asked about advantages and disadvantages of the MR technology, users gave many favorable responses (e.g., “exciting”, “very interesting”, and “fun”). The familiarization period was generally rated as quick (2).

The most frequently mentioned problem, reported by 22 (88%) participants, was visual impairment due to the graphical limitations of the virtual reconstructed reality. Three participants (12%) reported symptoms of cybersickness such as dizziness, nausea, and headache.

To improve the visual problems due to the low resolution, we included a digital, projective bisector mirror, which provided a high-resolution 2D picture of a certain area of interest. However, three participants stated they would not have used the mirror if the visualization had been sharp enough (2).

During the post-session interview, 21 (84%) rated the interaction and communication options as satisfactory and profitable and highlighted operational aspects such as moving monitors into sight and gesture-based interactions including drawing on and pointing at objects helping to act independently. The tactile distinguishability of the pointer’s control elements was criticized several times. The VR glasses were rated as comfortable by 18 participants (72%) but some perceived them as cumbersome to wear (5; 20%).

Seven (28%) participants reported that the MR session heightened their stress levels and noted the necessity for high levels of concentration. Four (16%) users expressed concerns about losing track in more complex situations involving multiple actors. These participants claimed that these feelings were triggered by the high amount of information at the beginning of the scenario and the use of unfamiliar technology.

## Discussion

This study compared the user acceptance and feasibility of a real-time MR teleconsultation system with a video call teleconsultation system in a simulated ICU environment.

Both systems received predominantly positive evaluations. Most users found the ArtekMed system easy to use, and perceived workload and TCT did not differ significantly from the simulated video call system. Although the usability score for the MR system was lower and only moderately acceptable, 20 (80%) users expressed interest in the future use of the ArtekMed system.

As previously described by our study group, the technical requirements of the ArtekMed system are complex and require trained computer scientists for the setup and maintenance [15, 20]. The seven interruptions caused by technical issues were related to software, user, or setup errors, but not to system limitations or poor graphical resolution. A complete reconstruction of a complex ICU workspace into a virtual duplicate with a sharp digital surface without holes is difficult to realize with currently available means (see Additional File K). Our previous studies using the ArtekMed system had already revealed these problems [15] and one proposed solution was the introduction of the digital bisectoral mirror into the operational features of the controller [28]. Users in our study rated the utility of the mirror as necessary but not very intuitive.

Similar problems were reported by Gasques et al. [18], who developed a comparable teleconsultation system, ARTEMIS—a real-time MR collaborative system for telementoring surgical procedures: the 3D point cloud was not capable of accurately representing detailed image sections of the surgical field, prompting the experts to use an additional camera image for detailed views.

In addition to a good graphical resolution, it is essential to precisely calibrate and synchronize the AR-HMD and the MR surrounding it. For example, inaccurate VR user annotations with a discrepancy of more than 1–2 cm or time aberrance due to delayed signal transmission are a pitfall for the feasibility of MR systems in healthcare. These and similar technical problems are common for real-time MR teleconsultation systems: The ARTEMIS study [18] and users of the STAR platform—another comparable teleconsultation system [14]—reported problems with the spatial inaccuracy of virtual annotations. Currently, when asked about the advantage regarding the speed of either system, 53% of the users prioritized the ArtekMed MR system over the control system. Regarding the feeling of security, only 42% favored the MR system. Uncertainty in the operational features affected the sense of security as many users explained in the post-session interview. The unfamiliar operation of the new system is reflected in the low SUS scores of the MR system.

Although facing similar challenges, Rojas-Muñoz et al. received better usability results for the STAR platform. In their clinical evaluation, ten surgical novices using the STAR platform as guidance made significantly fewer mistakes during a surgical intervention and reached higher confidence scores and procedural performance levels than the control group. However, the control group included participants receiving no external guidance. A comparison to another telementoring system is still pending for both systems, STAR and ARTEMIS.

### Limitations

However, this criticism can partially be applied to our study as well. The comparator of the ArtekMed MR teleconsultation system was not a mobile telemedical cart with bidirectional audiovisual signals, including multiple cameras with zoom and remote-control functions [5, 31], but a single, non-adjustable ceiling-mounted camera. Future work could include a comparison with such a modern telemedical cart, which is currently being tested in a network of Bavarian intensive care units [6].

The rating of immersion and feeling of physical presence proved that the ICU-experienced healthcare professionals found themselves in a realistic clinical environment. However, some nurses reported discomfort with the role of the remote expert, as we called them a “senior physician”. We, nevertheless, doubt that this shift of perspectives had any influence on the external validation of this study, as we informed the participants that the focus of the study was not on the correct medical treatment but on the usability of both systems.

Although the facilitator used a structured script to moderate the scenarios, certain suggestions of the participants may have caused the facilitator to deviate from the script, and thus may have compromised the internal validity of the study. The use of a single facilitator for all trial runs mitigated this potential bias.

### Outlook

Once familiarity issues are overcome, we anticipate improved usability acceptance of the ArtekMed system, as the interactive features were positively attributed. This implies that more users might opt for the MR system in the future.

Until then, it is important to provide a comprehensive structured introduction to the system’s operational features to ensure that all users have a mutual understanding of the other user’s perspective and all interaction possibilities (see Additional File L). To also evaluate the user-friendliness of the ArtekMed teleconsultation system from the perspective of the AR user, we conducted an additional comparative study with the same setup which we are about to analyze. Upon analysis of the results, we will have a comprehensive user assessment of the whole teleconsultation system. Furthermore, a clinical trial with two inexperienced study participants for both MR user perspectives should be performed to uncover further advantages and shortcomings of our teleconsultation system. Currently, there are no legal regulations on how medical MR systems are certified [32], but a summative evaluation might be the next step in the system development process.

Apart from the usability issues revealed in our study, ethical concerns, protection of patient data, cybersecurity issues, and the shortage of skilled technical personnel must be addressed.

## Conclusion

Collaboration in the ICU using a real-time MR teleconsultation system can be realized successfully. In the conducted study, perceived workload and TCT do not differ significantly between the two mentioned systems. The ArtekMed MR system reaches lower usability scores (SUS), which is mostly attributed to the incomplete virtual reconstruction of the ICU workplace leading to visual impairments: technical obstacles must be mitigated before further clinical implementation. Nevertheless, 80% of the study participants can envision incorporating the system into their daily work routine.

## Supplementary Information

Supplementary material 1.

## Data Availability

The data set used and analyzed in the current study are available from the corresponding author on reasonable request.

## Abbreviations

AR: Augmented reality
ArtekMed: Augmented reality teleconsultation in medicine
BMBF: German Federal Ministry of Education and Research
HMD: Head mounted display
ICU: Intensive care unit
INM: Institut für Notfallmedizin und Medizinmanagement
LMU: Ludwig-Maximilians-University Munich
MR: Mixed reality
NASA-TLX: NASA task load index
SUS: System usability scale
TCT: Task completion time
VR: Virtual reality
XR: Extended reality
