# Supplementary material for: Comparison of the ArtekMed mixed reality teleconsultation system with a standard video call system in critical care: user acceptance and feasibility analysis
Source: Intensive Care Med Exp. 2025 May 8;13:49. doi: 10.1186/s40635-025-00758-4 (PMC12058577; doi:10.1186/s40635-025-00758-4)

# **Additional Files**

Table of Content

[1 Additional Files 1](#_Toc198710277)

[2 Additional File A: Scenario Description 1](#_Toc198710278)

[3 Additional File B: Simulation station instruction 2](#_Toc198710279)

[4 Additional File C: Questionnaire NASA-TLX 3](#_Toc198710280)

[5 Additional File D: Questionnaire System-Usability-Scale (SUS) 4](#_Toc198710281)

[6 Additional File E: Interview Questions 5](#_Toc198710282)

[6.1 Pression Interview: Questionnaire Readiness for Technology 5](#_Toc198710283)

[6.2 Pre-Session Interview Questions 5](#_Toc198710284)

[6.3 Post-Session Interview 6](#_Toc198710285)

[7 Additional File F: Video Example ArtekMed 6](#_Toc198710286)

[8 Additional File G: Task Completion Time 7](#_Toc198710287)

[9 Additional File H: NASA-TLX 7](#_Toc198710288)

[10 Additional File I: Technical Interruptions 7](#_Toc198710289)

[11 Additional File J: Barchart Usefulness 8](#_Toc198710290)

[12 Additional File K: Figure 8](#_Toc198710291)

[13 Additional File L: Figure 9](#_Toc198710292)

# **Additional File A: Scenario Description**

The participants acted as remote experts in four different medical scenarios, all including an intubated, sedated ARDS patient, suffering from hypoxemia. The underlying reason is an atelectasis (Scenario A1), a misplaced ventilating tube in the right main bronchus (Scenario A2), an accidental extubation (Scenario B1) and a tension pneumothorax (Scenario B2).

| Background information | |
| --- | --- |
| A 57 years old female patient, suffering from COVID-19 acute respiratory distress syndrome (ARDS), was lying in a prone position for the last 16 hours. She is sedated (RAAS-3) and mechanically ventilated via endotracheal tube (pressure controlled ventilation: PEEP 10mbar; P control 10mbar; respiratory rate 22/min, fraction of oxygen 50%).  Shortly after the turning maneuver, the local staff recognizes the patient is hypoxic with an oxygen saturation of only 83%. | |
| Scenario A1 | Problem: The problem lying underneath is an atelectasis on the lower right side of the lung.  Solution: Enhancing the pressure controlled ventilation, for example by raising PEEP and/or P control. |
|  |  |
| Scenario A2 | Problem: The problem lying underneath the hypoxia is the dislocation of the endotracheal tube into the right main bronchus  Solution: Repositioning of the endotracheal tube. |
|  |  |
| Scenario B1 | Problem: While turning the patient on her back, the endotracheal tube was dislocated. It is no longer possible to ventilate via endotracheal tube. The bedside staff is successfully performing bag-mask ventilation. The oxygen saturation remains at 83-85%. Wakening the patient is not possible as the ARDS stage still requires invasive ventilation treatment and the patient just received a muscle relaxant before starting the maneuver  Solution: Reintubation or other way of securing the airway |
|  |  |
| Scenario B2 | Problem: After a recruitment maneuver, the oxygen saturation fell again to 83-85%. In addition, heart rate raised to 140/min and RR measures 90/45 mmHg while the norepinephrine dose was already doubled (1.6 mg/h). There is no breathing sound on the left side of the torso.  Solution: Needle decompression of the tension pneumothorax |
|  |  |

# **Additional File B: Simulation station instruction**

Experienced technicians of the INM’s Human Simuation Center controlled the Simulation Station They followed a detailed script, describing the reactions of the simulator to the actions performed by the local staff. The script was structured in phase 1-4. For each phase, the situation and the problem were explained and the corresponding vital signs and settings of the ventilation simulation (Hamilton C6 Sim) were advised. The following table shows the script of Scenario A1.

| Scenario A1 | Phase 1 | Phase 2 | Phase 3 | Phase 4 |
| --- | --- | --- | --- | --- |
| Situation | Instructor explains reason for consultation | If FiO2 was raised | If a sedation bolus was given | Solution found, treatment was ordered |
| Problem | Atelectasis in the right lower lung area |  |  | Atelectasis treated with increasing pressure and PEEP |
| Vital signs | Eyes closed  spO2 83%  RR 98/45  HR 110  no CO2 | spo2 88% | RR 90/40 | spO2 95%  RR 110/60  HR 90 |
| Ventilation Simulation  *Hamilton C6 Sim*  -ARDS2  -Female 168cm  -Resistance and  Compliance no changes to standard setting  -Metabolism  DecReased  >Start ventilation | PCV+  BR 22  P control 10  PEEP 10  FiO2 0,5  I:E 1:3  Co2/spO2 auto | FiO2 … |  | Instruction of remote expert |

# Additional File C: Questionnaire NASA-TLX

Please indicate for each of the dimensions below how much workload you experienced. Mark on the following scales the degree to which you felt challenged or stressed in the six mentioned dimensions during the task.

**Mental Demand**

How much mental and perceptual activity - thinking, deciding, calculating, searching...)? Was the task easy or demanding, simple or complex, exacting or forgiving?

|  |  |  |  |  |  |  |  |  |  |  |  |  |  |  |  |  |  |  |  |
| --- | --- | --- | --- | --- | --- | --- | --- | --- | --- | --- | --- | --- | --- | --- | --- | --- | --- | --- | --- |
|  |  |  |  |  |  |  |  |  |  |  |  |  |  |  |  |  |  |  |  |
| Very Low | | | |  |  |  |  |  |  |  |  |  |  |  |  | Very High | | | |

**Physical Demand**

How much physical activity was required - pulling, pushing, turning? Was the task easy or demanding, slow or brisk, restful or laborious?

|  |  |  |  |  |  |  |  |  |  |  |  |  |  |  |  |  |  |  |  |
| --- | --- | --- | --- | --- | --- | --- | --- | --- | --- | --- | --- | --- | --- | --- | --- | --- | --- | --- | --- |
|  |  |  |  |  |  |  |  |  |  |  |  |  |  |  |  |  |  |  |  |
| Very Low | | | |  |  |  |  |  |  |  |  |  |  |  |  | Very High | | | |

**Temporal Demand**

How much time pressure did you feel? Was the pace slow and leisurely, or rapid and frantic?

|  |  |  |  |  |  |  |  |  |  |  |  |  |  |  |  |  |  |  |  |
| --- | --- | --- | --- | --- | --- | --- | --- | --- | --- | --- | --- | --- | --- | --- | --- | --- | --- | --- | --- |
|  |  |  |  |  |  |  |  |  |  |  |  |  |  |  |  |  |  |  |  |
| Very Low | | | |  |  |  |  |  |  |  |  |  |  |  |  | Very High | | | |

**Performance**

How successful do you feel in accomplishing the goals of the task? How satisfied were you with your performance?

|  |  |  |  |  |  |  |  |  |  |  |  |  |  |  |  |  |  |  |  |
| --- | --- | --- | --- | --- | --- | --- | --- | --- | --- | --- | --- | --- | --- | --- | --- | --- | --- | --- | --- |
|  |  |  |  |  |  |  |  |  |  |  |  |  |  |  |  |  |  |  |  |
| gut | | | |  |  |  |  |  |  |  |  |  |  |  |  | schlecht | | | |

**Effort**

How hard did you work mentally and physically to accomplish your level performance?

|  |  |  |  |  |  |  |  |  |  |  |  |  |  |  |  |  |  |  |  |
| --- | --- | --- | --- | --- | --- | --- | --- | --- | --- | --- | --- | --- | --- | --- | --- | --- | --- | --- | --- |
|  |  |  |  |  |  |  |  |  |  |  |  |  |  |  |  |  |  |  |  |
| Very Low | | | |  |  |  |  |  |  |  |  |  |  |  |  | Very High | | | |

**Frustration**

How discouraged, irritated, stressed, and annoyed versus gratified, confident, relaxed, and content did you feel during the task?

|  |  |  |  |  |  |  |  |  |  |  |  |  |  |  |  |  |  |  |  |
| --- | --- | --- | --- | --- | --- | --- | --- | --- | --- | --- | --- | --- | --- | --- | --- | --- | --- | --- | --- |
|  |  |  |  |  |  |  |  |  |  |  |  |  |  |  |  |  |  |  |  |
| Very Low | | | |  |  |  |  |  |  |  |  |  |  |  |  | Very High | | | |

# Additional File D: Questionnaire System-Usability-Scale (SUS)

1. I think that I would like to use this system frequently.

| Strongly disagree  1 | 2 | 3 | 4 | Strongly agree  5 |
| --- | --- | --- | --- | --- |

1. I found the system unnecessarily complex.

| Strongly disagree  1 | 2 | 3 | 4 | Strongly agree  5 |
| --- | --- | --- | --- | --- |

1. I thought the system was easy to use.

| Strongly disagree  1 | 2 | 3 | 4 | Strongly agree  5 |
| --- | --- | --- | --- | --- |

1. I think that I would need the support of a technical person to be able to use this system.

| Strongly disagree  1 | 2 | 3 | 4 | Strongly agree  5 |
| --- | --- | --- | --- | --- |

1. I found the various functions in this system were well integrated.

| Strongly disagree  1 | 2 | 3 | 4 | Strongly agree  5 |
| --- | --- | --- | --- | --- |

1. I thought there was too much inconsistency in this system.

| Strongly disagree  1 | 2 | 3 | 4 | Strongly agree  5 |
| --- | --- | --- | --- | --- |

1. I would imagine that most people would learn to use this system very quickly.

| Strongly disagree  1 | 2 | 3 | 4 | Strongly agree  5 |
| --- | --- | --- | --- | --- |

1. I found the system very cumbersome to use.

| Strongly disagree  1 | 2 | 3 | 4 | Strongly agree  5 |
| --- | --- | --- | --- | --- |

1. I felt very confident using the system.

| Strongly disagree  1 | 2 | 3 | 4 | Strongly agree  5 |
| --- | --- | --- | --- | --- |

1. I needed to learn a lot of things before I could get going with this system.

| Strongly disagree  1 | 2 | 3 | 4 | Strongly agree  5 |
| --- | --- | --- | --- | --- |

# Additional File E: Interview Questions

## Pression Interview: Questionnaire Readiness for Technology

1. I am very curious about new technological developments.

| Strongly disagree  1 | Disagree  2 | Neither agree or disagree  3 | Agree  4 | Strongly agree  5 |
| --- | --- | --- | --- | --- |

1. I quickly take a liking to new technological developments.

| Strongly disagree  1 | Disagree  2 | Neither agree or disagree  3 | Agree  4 | Strongly agree  5 |
| --- | --- | --- | --- | --- |

1. I am always interested in using the latest technological devices.

| Strongly disagree  1 | Disagree  2 | Neither agree or disagree  3 | Agree  4 | Strongly agree  5 |
| --- | --- | --- | --- | --- |

1. If I had the opportunity, I would use technological products much more frequently than I currently do.

| Strongly disagree  1 | Disagree  2 | Neither agree or disagree  3 | Agree  4 | Strongly agree  5 |
| --- | --- | --- | --- | --- |

## Pre-Session Interview Questions

1. To what extent are you familiar with working in the intensive care unit?
2. How often do you conduct rounds/treatments in the intensive care unit (number per month)?
3. How often does it happen in your daily work that you are asked for advice without being physically present?
4. Have you had any experience with teleconsultation before? If yes, in what context?
5. If yes, how do you conduct teleconsultation? (Phone call, SMS, WhatsApp, Skype)
6. How many hours per week do you use digital media (both privately and professionally)? (Including social media, PC, and mobile devices)
7. How many hours per week do you spend playing video games?
8. Are you familiar with the terms "Virtual Reality" and "Augmented Reality"?
9. How often have you used a Virtual Reality system before?
10. How many hours per week do you use Virtual Reality systems?
11. How often have you used an Augmented Reality system before?
12. Please indicate your opinion on the following statement: “I think VR and AR will improve medical treatment in the future”

| No  1 | Rather no  2 | Unsure  3 | Rather yes  4 | Yes  5 |
| --- | --- | --- | --- | --- |

## Post-Session Interview

1. (In general) How was the test?
2. How would you rate the communication with the nurse?
3. With which system did you feel safer?
4. With which system were you faster?
5. What is your overall impression of the AR/VR system? (Point out particularly positive/negative aspects)
6. How did you find the activity in the 3D environment? Were you able to immerse yourself in the scenario?
7. How would you rate your adaptation time?
8. How did it feel to wear the VR headset?
9. How would you rate the image and sound quality?
10. Did you experience any symptoms during the task that are commonly associated with 3D simulations (dizziness, nausea)?
11. How would you rate the interaction possibilities (drawing, pointing)?
12. If the participant did not mention the annotations yet: Were these helpful? What could be improved about them?
13. How do you rate the use of the "mirror"?
14. How could the system be improved from your perspective?
15. Can you imagine using VR teleconsultation in your daily work in the intensive care unit? In what cases/scenarios?
16. Please indicate your opinion on the following statement once more: “I think VR and AR will improve medical treatment in the future”

| No  1 | Rather no  2 | Unsure  3 | Rather yes  4 | Yes  5 |
| --- | --- | --- | --- | --- |

# Additional File F: Video Example ArtekMed

<https://owncloud.inm-online.de/index.php/s/hHc8r7eseij7ah1>

# Additional File G: Task Completion Time

| Scenario | N | Video call Mean (mm:ss) | SD | Mixed Reality Mean (mm:ss) | SD | T-Test; [95% confidence interval]; p-value |
| --- | --- | --- | --- | --- | --- | --- |
| A1 | 13/12 | 07:26 | 01:23 | 08:17 | 01:50 | -1.3; [-02:12;00:29]; p = 0.2 |
| A2 | 12/13 | 06:43 | 01:57 | 07:42 | 01:24 | -1.4; [-02:22;00:25]; p = 0.2 |
| B1 | 12/13 | 07:11 | 01:34 | 09:03 | 01:36 | -3.0; [-03:27;-00:41]; p = 0.007 |
| B2 | 13/12 | 08:07 | 02:34 | 08:35 | 02:10 | -0.5; [-02:27;01:30]; p = 0.6 |

# Additional File H: NASA-TLX

| Scenario | N | Video call  Mean | Mixed Reality  Mean | T-Test; [95% confidence interval]; p-value |
| --- | --- | --- | --- | --- |
| A1 | 13/12 | 39.4 | 44.8 | -1.0; [-16.6; 5.9]; p = 0.3 |
| A2 | 12/13 | 37.5 | 41.9 | -0.7; [-16.7; 7.9]; p = 0.5 |
| B1 | 12/13 | 45.2 | 47.2 | -0.4; [-12.7; 8.7]; p = 0.7 |
| B2 | 13/12 | 45.9 | 62.9 | -2.9; [-29.1;-4.9]; p = 0.008 |

# Additional File I: Technical Interruptions

|  | Problem | Classification | Solution | File | Start [h:mm:ss] | End [h:mm:ss] |
| --- | --- | --- | --- | --- | --- | --- |
| 1 | Mirror function not available | Software malfunction | Unity restart required | 2022-12-05_08-32-36-MUE07MA_A1_VR | 0:03:05,6 | 0:04:20,3 |
| 2 | Mirror function not available | User-related problem | User instruction | 2022-12-12_15-27-53-GR08TA_A1_VR | 0:03:57,9 | 0:05:38,2 |
| 3 | Improperly fitted VR HMD | User-related problem | User instruction | 2022-11-25_15-38-15-MA84RO_A2_VR | 0:00:57,8 | 0:01:31,3 |
| 4 | Audio connection inactive | Hardware setup incomplete | Start Skype call and select speaker | 2022-12-19_17-28-52-DE13CH_A2_VR | 0:00:26,0 | 0:01:01,4 |
| 5 | Setting error in ventilator simulation | not ArtekMed related |  | 2022-11-10_14-54-50-DA22RE_A2_VR | 0:05:56,8 | 0:06:25,6 |
| 6 | Mirror function not available | Software malfunction | Unity restart required | 2022-12-15_08-46-06-PE75KA_B2_VR | 0:06:28,0 | 0:08:12,5 |
| 7 | HoloLens HMD malfunction | Software malfunction | HoloLens HMD restart required | 2022-11-21_15-56-59-LI73AN_B2_VR | 0:00:33,0 | 0:02:14,3 |

# Additional File J: Barchart Usefulness

Opinion on the usefulness of VR & AR systems (pre- and post-session answers)


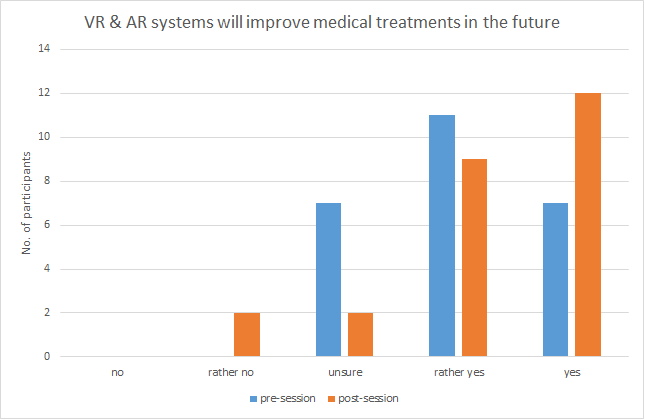


# Additional File K: Figure

The figure visualizes the improvement of pixilation by using the digital mirror, which offers a 2D image of a certain area of interest.


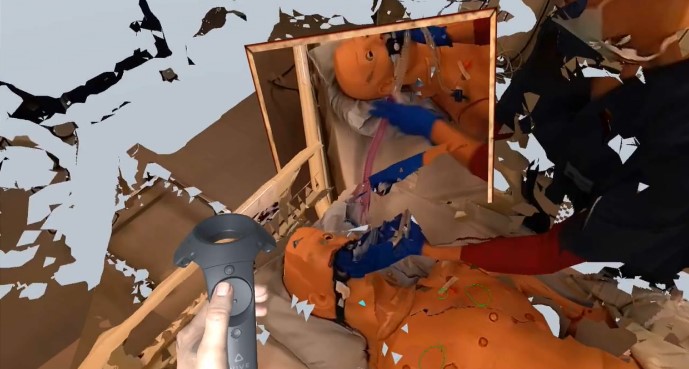


# Additional File L: Figure

The VR user’s view with holes in the virtual reconstructed scene. The green annotations on the mannequin’s chest mark the area of lung auscultation, which were added by the VR user.


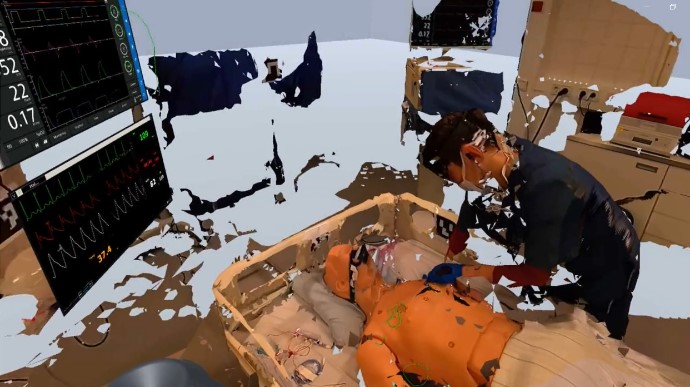

Supplement: Supplementary file 1 — Supplementary material 1. [file 40635_2025_758_MOESM1_ESM.docx]
